# Modeling insurance claims using Bayesian nonparametric regression

**DOI:** 10.1371/journal.pone.0346734

**Published:** 2026-04-10

**Authors:** Mostafa Shams, Kaushik Ghosh

**Affiliations:** 1 Department of Statistical Sciences, Wake Forest University, Winston-Salem, North Carolina, United States of America; 2 Department of Mathematical Sciences, University of Nevada, Las Vegas, Nevada, United States of America; ICAR-CNR: Istituto di Calcolo e Reti ad Alte Prestazioni Consiglio Nazionale delle Ricerche, ITALY

## Abstract

Predicting future insurance claims using observed covariates is essential for actuaries in setting appropriate insurance premiums. For this purpose, actuaries commonly employ parametric regression models, which assume the same functional form tying the response to the covariates across all data points. However, these models may lack the flexibility required to accurately capture, at the individual level, the relationship between covariates and claims frequency and severity. This limitation is particularly relevant as claims data are often multimodal, highly skewed, and heavy-tailed. In this paper, we explore the use of Bayesian nonparametric (BNP) regression models to predict claims frequency and severity based on covariates. Specifically, we model claims frequency as a mixture of Poisson regression and the logarithm of claims severity as a mixture of normal regression. We then employ Dirichlet process (DP) and Pitman–Yor process (PY) as priors for the mixing distribution over the regression parameters. Unlike parametric regression, such models allow each data point to have its own individual parameters, thereby making them highly flexible and resulting in improved prediction accuracy. We describe model fitting using Markov chain Monte Carlo (MCMC) methods and illustrate their applicability using two independent real-world insurance datasets. The proposed BNP models reduced the mean squared error for the French and Belgian claims frequency data by approximately 52% and 33%, respectively (relative to standard Poisson regression), and for the corresponding claims severity data by nearly 45% and 79%, respectively (relative to standard multiple linear regression).

## 1 Introduction

Insurance claim datasets contain two parts: the claims frequency indicating the number of claims and the claims severity indicating the monetary amount of each claim. In modeling insurance claims, frequency and severity are often modeled separately using a variety of statistical methods. A common approach involves parametric regression models, such as Generalized Linear Models (GLMs), which assume a fixed functional form between the covariates and the response variable (see, for example, [1,2]). While widely used, these models can be limited when applied to insurance data, which often exhibit complex characteristics such as multimodality, severe right-skewness, and heavy tails that are not well-captured by a single, global distribution. This can lead to a lack of flexibility and, consequently, to less accurate predictions.

To address these limitations, actuaries and statisticians have explored more flexible alternatives. Semi-parametric models, such as Generalized Additive Models (GAMs), utilize splines to capture non-linear relationships, while penalized regression models, like those using Lasso or Ridge regularization, handle high-dimensional covariate spaces. Quantile regression has also been applied to better characterize the conditional distribution of claims beyond the mean, making it particularly useful for heavy-tailed insurance data. These methods allow greater flexibility but still depend on choices about smoothing, penalties, or quantile levels, which can affect their performance.

Several studies have demonstrated these advances. [3] showed that extending GLMs to account for dependence between claim frequency and severity provides more realistic risk modeling compared to assuming independence. In a related direction, [4] introduced mixture composite regression models with feature selection, which offer a way to handle multimodality and improve prediction accuracy. In the area of semi-parametric modeling, [5] demonstrated that Bayesian GAMs can flexibly capture nonlinear effects across multiple distributional components, such as location, scale, and shape. Earlier, [6] highlighted the value of Bayesian GAMs in ratemaking, showing how spline-based methods reveal important nonlinear relationships in insurance data. Penalized regression methods, such as the Lasso, have also been applied in insurance to identify key predictors in high-dimensional settings, as shown in recent work on lapse rates [7]. More recently, quantile regression methods have been extended with machine learning tools, as [8] illustrated by applying neural networks for quantile-based claim amount estimation, providing a more accurate view of heavy-tailed risks.

More recent advancements have also seen the application of machine learning techniques, as well as Bayesian machine learning methods such as Gaussian processes and Bayesian deep learning, to insurance and risk prediction tasks. For example, in the area of claim severity, [9] introduced gamma mixture density networks, a deep learning approach that models heavy-tailed claim amounts more accurately than traditional methods. On the frequency side, [10] introduced Bayesian CART (Classification and Regression Trees) models for claims frequency, offering probabilistic insights and improved prediction compared to classical GLMs. Beyond Bayesian approaches, boosting methods have also gained traction: [11] developed a stochastic gradient boosting frequency–severity model, showing clear performance gains, while [12] applied gradient boosting to motor insurance, demonstrating its usefulness in modeling both frequency and severity of claims.

We often aim to model insurance claims as a function of covariates, which leads to a regression problem. One issue with parametric regression models is that they assume a fixed response to covariates, with each data point sharing the same regression parameters. Bayesian nonparametric regression models, on the other hand, allow each data point to have individualized regression parameters. This paper explores Bayesian nonparametric (BNP) regression models, specifically the Dirichlet process mixture model (DPMM) and the Pitman–Yor process mixture model (PYMM), for modeling claims frequency and severity, and predicting future insurance losses.

These Bayesian nonparametric regression models offer greater flexibility and more accurate predictions compared to traditional parametric regression. They can capture complex and non-standard distributions of insurance claims, thereby improving the accuracy of future claim predictions. Furthermore, they can identify latent clustering structures within the data. In BNP regression, we treat the insurance claim distribution itself as an unknown parameter, which means we select a prior distribution for the probability distributions. Consequently, a key advantage of BNP regression over Bayesian parametric regression models is the flexibility to incorporate uncertainty at the level of distribution functions.

Research on the application of Bayesian nonparametric regression for insurance loss data is limited. For example, Dirichlet process mixture models for insurance loss data have been discussed in [13–18]. However, [14–16] focused on density estimation without incorporating covariates, while [17,18] considered regression but only for claim severity. Our work builds upon and extends this existing body of literature by presenting a BNP regression framework for both components of insurance claims data. The novelty of this study is that we compare both Dirichlet process (DP) and Pitman–Yor process (PY) priors in regression settings for claim frequency and severity, and we evaluate their prediction accuracy against standard parametric, semi-parametric, and penalized regression models.

The remainder of this paper is organized as follows. Section 2 reviews two commonly used BNP models: the Dirichlet process mixture model and the Pitman–Yor process mixture model. In Section 3, we present the models for predicting claims frequency, and we report the corresponding results based on real insurance claims frequency data in Section 4. We then introduce the models for claims severity in Section 5, followed by Section 6, where we summarize the results from the claims severity analysis. Finally, in Section 7, we provide concluding remarks and discuss potential directions for future research. A summary of the mathematical notation used in this paper is available in the supporting information section (see S1 Table).

## 2 Bayesian nonparametric models

### 2.1 Dirichlet process mixture model (DPMM)

The Dirichlet process (DP), introduced by [19], provides a nonparametric prior for probability distributions. It is denoted by $\text{DP}(\alpha ,G_{0})$ and has two parameters: a scalar precision parameter α>0 and a base probability measure *G*_0_. As a consequence of the stick-breaking representation, proved by [20], the Dirichlet process generates distributions that are discrete with probability 1. To model continuous phenomena, DP can be used as a prior for the mixing distribution over the parameters of a distribution. This leads to Dirichlet process mixture models (DPMM), which can be expressed as a hierarchical model:


$$\begin{array}{ccc} & & y_{i}|\theta_{i}\overset{\mathrm{ind.}}{∼}F(\theta_{i})\;;\;i=1,\ldots ,n\\ & & \theta_{i}|G\overset{\mathrm{iid}}{∼}G\;;\;i=1,\ldots ,n\\ & & G\sim \text{DP}(\alpha ,G_{0}). \end{array}$$

From a computational point of view, [21] has presented several Markov chain Monte Carlo (MCMC) algorithms for sampling from the posterior distribution of DPMM. It can be shown that if we integrate over *G* in the BNP model above, we obtain the following Pólya urn predictive rule:


$$\theta_{i}|\theta^{\left(-i\right)}\sim \frac{1}{n-1+\alpha }\sum_{j\neq i}\delta_{\theta_{j}}(.)+\frac{\alpha }{n-1+\alpha }G_{0},$$ (1)

where *n* is the number of observations, $\delta_{\theta }(.)$ is the distribution concentrated at the single point θ, and $\theta^{\left(-i\right)}=(\theta_{j}:j\neq i)$. This leads to a Gibbs sampling algorithm to draw posterior samples in DPMM. When the base probability measure *G*_0_ is non-conjugate for the likelihood (*F*) of the model, this Gibbs sampling is not computationally feasible. [21] presents a Gibbs sampling method with auxiliary variables called Neal’s Algorithm 8 for models with non-conjugate prior. This sampling method can also be used for models with conjugate prior in order to avoid computing integrals $\int g_{0}(\theta )f(y_{i}|\theta )\;d\theta$ for conditional probabilities within the MCMC algorithm (*g*_0_ and *f* are probability functions for distributions *G*_0_ and *F* respectively). In Neal’s Algorithm 8, the Gibbs sampling is applied to the vectors $c=(c_{1},\ldots ,c_{n})$ called configuration (or clustering) vectors, where *c*_*i*_ is an integer indicating the cluster label associated with the data point *y*_*i*_ (see [21]).

The critical parameter for DPMM is the precision parameter α of the DP prior, controlling the variance and the level of clustering, indicating that the larger α results in *G* which is closer to the parametric base distribution *G*_0_ and therefore larger number of clusters. In this paper, we place a gamma prior on the DPMM’s precision parameter α. Building on [22], who derived the conditional distribution of the number of distinct components (*K*) given α, [23] developed an efficient method to update α at each MCMC iteration. We use this approach to update α for our DPMM.

### 2.2 Pitman–Yor process mixture model (PYMM)

The Pitman–Yor process (PY), denoted by $\text{PY}(d,\alpha ,G_{0})$, is a generalization of the Dirichlet process. It offers greater flexibility over tail behavior than the Dirichlet process, which exhibits exponential tails. The PY is parameterized by a discount parameter 0 ≤ *d* < 1, a strength parameter α>−d, and a base probability measure *G*_0_. Setting *d* = 0, the Pitman–Yor process becomes $\text{DP}(\alpha ,G_{0})$. Pitman–Yor process has a heavier tail than the Dirichlet process. Since many real-world distributions such as insurance loss data have distributions with heavier tails than exponential, this makes the Pitman–Yor process be possibly a better choice for a prior over mixing distribution in mixture models than Dirichlet process (see [24–27]).

Similar to Dirichlet process, Pitman–Yor process generates distributions that are discrete with probability 1. PY can be used as a prior for the mixing distribution over the parameters of one distribution. This leads to the Pitman–Yor process mixture models (PYMM), also known as the two-parameter Poisson-Dirichlet process mixture models, which can be written as a hierarchical model:


$$\begin{array}{ccc} & & y_{i}|\theta_{i}\overset{\mathrm{ind.}}{∼}F(\theta_{i})\;;\;i=1,\ldots ,n\\ & & \theta_{i}|G\overset{\mathrm{iid}}{∼}G\;;\;i=1,\ldots ,n\\ & & G\sim \text{PY}(d,\alpha ,G_{0}). \end{array}$$

It can be shown that if we integrate over *G* in the BNP model above, we obtain the following Pólya urn predictive rule for PY, which can be used to develop a Gibbs sampling algorithm to draw posterior samples in PYMM (see [25,27]).


$$\theta_{i}|\theta^{\left(-i\right)}\sim \sum_{j=1}^{K_{n-1}}\frac{n_{j}-d}{n-1+\alpha }\delta_{\theta_{j}^{*}}(.)+\frac{\alpha +dK_{n-1}}{n-1+\alpha }G_{0},$$ (2)

where $\theta^{\left(-i\right)}=(\theta_{j},j\neq i)$, *K*_*n*−1_ is the number of distinct components among $\theta^{\left(-i\right)}$, and $\theta_{j}^{*}$ (*j* = 1, …, *K*_*n*−1_) are the unique values among $\theta^{\left(-i\right)}$, and *n*_*j*_ is the frequency of $\theta_{j}^{*}$.

The MCMC algorithm for sampling from the PYMM is similar to Neal’s Algorithm 8 for the DPMM. However, differences arise in the conditional probabilities in the first step of Neal’s Algorithm 8. According to the Pólya urn predictive rule for PYMM, the configuration cluster label *c*_*i*_ in the first step of the Neal’s Algorithm 8 is updated by drawing new values from {1, …, *h*} using the conditional probabilities below (see [21,27]).


$$\begin{array}{c} P(c_{i}=c|c^{\left(-i\right)},y_{i},\phi_{1},\ldots ,\phi_{h})\;\propto \;\left\{ \begin{array}{cc} \left(n_{-i,c}-d)F(y_{i},\phi_{c}\right) & \text{for}\;\;\;1\;\leq \;c\;\leq \;K_{n}^{-}\\ \left((\alpha +dK_{n}^{-})/m)F(y_{i},\phi_{c}\right) & \text{for}\;\;\;K_{n}^{-}<c\leq h, \end{array}\right. \end{array}$$

where $n_{-i,c}$ is the number of *c*_*j*_ for *j* ≠ *i* that are equal to *c* and $K_{n}^{-}$ is the number of current distinct components among the observations except the observation *i*.

The PYMM’s discount parameter *d* and strength parameter α are crucial. We assign a uniform prior to *d* and a log-normal prior to (α+d) to ensure its non-negativity. Let *K*_*n*_ be a random variable that indicates the current number of distinct components at each iteration of the MCMC sampling algorithm. To determine the conditional posterior distributions of *d* and α and then update them at each MCMC iteration, we leverage the conditional distribution of *K*_*n*_ given both *d* and α, as established in [28]. We then employ a random-walk Metropolis-Hastings algorithm to sample from these posteriors. To achieve efficient mixing in random-walk Metropolis-Hastings, tuning associated proposal variances is crucial, as noted in [29]. Thus, we employ the Adaptive Metropolis-within-Gibbs method from the same work to update these variances at each MCMC iteration.

### 2.3 Clustering property of BNP models

As stated in the previous sections, Dirichlet process and Pitman–Yor process generate distributions that are discrete with probability 1. Therefore, the probability measure *G* in the BNP models above is a discrete probability measure, and this implies a positive probability for the ties among the parameters $\theta_{1},\theta_{2},\ldots ,\theta_{n}$. We can use these ties to define clusters, i.e., *K* < *n* distinct values of $\theta_{i}$’s, that induce a clustering structure on the dataset.

We assess the posterior clustering performance of our BNP regression models in our real data application. Let’s assume that there are *n* data points in our training dataset and our model’s regression parameter vectors are $\theta_{1},\ldots ,\theta_{n}$ where $\theta_{i}$ is the regression parameter vector related to the data point *i*. We then perform clustering based on samples from the posterior distribution of these parameter vectors. Clustering requires some methods for computing the dissimilarity between each pair of observations. We construct an *n* × *n* dissimilarity matrix using samples from the posterior distribution of $\theta_{1},\ldots ,\theta_{n}$. Each element in this matrix, specifically the entry in the *i*th row and *j*th column, represents the proportion of posterior samples where observations *i* and *j* are assigned different regression parameter vectors (i.e., posterior samples for $\theta_{i}$ and $\theta_{j}$ are different).

Unlike the traditional clustering algorithms, DPMM and PYMM do not require us to set the number of clusters *K* in advance. These two models define a mixture model with countably infinitely many components and can infer *K* from the data and allow *K* to grow as more data are collected.

## 3 Modeling claims frequency

When building an insurance pricing model, the first step is predicting claims frequency–that’s the number of claims. Actuaries model this frequency based on various covariates, known as risk factors in the insurance world. Let’s assume that there are *n* insurance policies with a set of *k* covariates for each. The *i*th policy’s covariates are denoted by the vector $x_{i}=(1,x_{i1},\ldots ,x_{ik}{)}^{T}$. The *i*th policy’s recorded number of claims is denoted by $y_{i}\in \{0,1,2,\ldots \}$. In order to determine the size of potential losses in any type of insurance, one must also know the corresponding exposure. We let *t*_*i*_ represent the length of time or exposure for the *i*th policy. The parametric Poisson regression model *t*hat has been widely used to model *y*_*i*_ is


$$y_{i}|\beta \;\overset{\mathrm{ind.}}{∼}\text{ Poisson}(t_{i}\exp(x_{i}^{T}\beta ))\;;\;i=1,\ldots ,n,$$

where $\beta =(\beta_{0},\beta_{1},\ldots ,\beta_{k}{)}^{T}$ is the vector of regression coefficients. However, the model above assumes that each data point has the same regression parameter vector, β, and thus similar response to covariates for each individual. In this section, we use DPMM and PYMM which allow each data point to have its own regression parameter vector. Due to the clustering property of DP and PY priors, the data points are then clustered by their shared regression parameters (see [13,30]).

### 3.1 Model description

The BNP Poisson regression parameters here are the regression coefficient vectors, $\beta_{i}=(\beta_{i0},\beta_{i1},\ldots ,\beta_{ik}{)}^{T}$, for i=1,…,n. We propose two BNP models for the number of claims data as follows.

First, we model the number of claims, *y*_*i*_, using a Dirichlet process mixture of Poisson regression, by setting a DP prior on the mixing distribution over the regression parameters, $\beta_{i}$. Additionally, a Gamma prior is placed on the precision parameter α. This DPMM can be expressed hierarchically as:


$$\begin{array}{cc} & y_{i}|\beta_{i}\;\overset{\mathrm{ind.}}{∼}\text{ Poisson}(t_{i}\exp(x_{i}^{T}\beta_{i}))\;;\;i=1,\ldots ,n\\ & \beta_{i}|G\;\overset{\mathrm{iid}}{∼}\;G\;;\;i=1,\ldots ,n\\ & G\;\sim \text{ DP}(\alpha ,G_{0})\\ & G_{0}\;=\text{ MN}(0_{k+1},I_{k+1})\\ & \alpha \;\sim \text{ Gamma}(1,1). \end{array}$$ (3)

Here, the base probability measure *G*_0_ is taken to be a multivariate normal distribution with a (*k* + 1)-dimensional mean vector $0_{k+1}$ and a (*k* + 1) × (*k* + 1) covariance matrix *I*_*k*+1_ where *I*_*k*+1_ is the identity matrix of size *k* + 1.

Alternatively, we set a PY prior instead of a DP prior on the mixing distribution over the regression parameters, $\beta_{i}$, and model *y*_*i*_ using a Pitman–Yor process mixture of Poisson regression. In this case, we place a uniform prior on the discount parameter *d* and a log-normal prior on the sum of the precision parameter and discount parameter, (α+d), ensuring its non-negativity. Our PYMM can then be written as a hierarchical model:


$$\begin{array}{cc} & y_{i}|\beta_{i}\overset{\mathrm{ind.}}{∼}\text{ Poisson}(t_{i}\exp(x_{i}^{T}\beta_{i}))\;;\;i=1,\ldots ,n\\ & \beta_{i}|G\;\overset{\mathrm{iid}}{∼}\;G\;;\;i=1,\ldots ,n\\ & G\;\sim \text{ PY}(d,\alpha ,G_{0})\\ & G_{0}\;=\text{ MN}(0_{k+1},I_{k+1})\\ & d\sim \text{Uniform}(0,1)\\ & (\alpha +d)|d\sim \text{Log-Normal}(0,1). \end{array}$$ (4)

### 3.2 Computation of posterior predictive distributions

The posterior predictive distribution for the future number of claims, *y*_*n*+1_, represents the conditional probability mass function of *y*_*n*+1_ given the observed data (*y*_1_, …, *y*_*n*_). This prediction implicitly assumes a new covariate vector $x_{n+1}$ and a new exposure *t*_*n*+1_. Thus, our notation $f(y_{n+1}|y_{1},\ldots ,y_{n})$ is implicitly equivalent to


$$f\left(y_{n+1}|y_{1},\ldots ,y_{n}\right)=f\left(y_{n+1}|y_{1},\ldots ,y_{n},x_{n+1},t_{n+1}\right).$$

For DPMM, the parameters are the coefficients of the BNP Poisson regression, $\beta_{i}$. The posterior predictive distribution is obtained by integrating over the parameters. By conditional independence, *y*_*n*+1_ depends only on its own parameter $\beta_{n+1}$, and it follows that


$$\begin{array}{cc} f(y_{n+1}|y_{1},\ldots ,y_{n}) & =\int \cdots \int f(y_{n+1}|\beta_{n+1})\times f(\beta_{n+1}|\beta_{1},\ldots ,\beta_{n},\alpha )\\ & \;\times f(\beta_{1},\ldots ,\beta_{n},\alpha |y_{1},\ldots ,y_{n})\;d\beta_{n+1}\;d\beta_{n}\ldots \;d\beta_{1}\;d\alpha . \end{array}$$

Using the Pólya urn predictive rule of DP in Eq (1), it can be shown that


$$\begin{array}{cc} f\left(y_{n+1}|y_{1},\ldots ,y_{n}\right) & =\left(\int \frac{\alpha }{\alpha +n}f\left(\beta_{1},\ldots ,\beta_{n},\alpha |y_{1},\ldots ,y_{n}\right)\;d\beta_{n}\ldots \;d\beta_{1}\;d\alpha \right)\\ & \times \left(\int f(y_{n+1}|\beta_{n+1})g_{0}(\beta_{n+1})\;d\beta_{n+1}\right)+\int \cdots \int \frac{1}{\alpha +n}\sum_{j=1}^{n}f\left(y_{n+1}|\beta_{j}\right)\\ & \times f\left(\beta_{1},\ldots ,\beta_{n},\alpha |y_{1},\ldots ,y_{n}\right)\;d\beta_{n}\ldots \;d\beta_{1}\;d\alpha . \end{array}$$ (5)

where *g*_0_ is the probability density function for the base probability distribution *G*_0_. By getting *M* samples from the posterior distribution of $\left(\beta_{1},\ldots ,\beta_{n}\right)$ and α, with the *m*^th^ sample being $\left(\beta_{1}^{\left(m\right)},\ldots ,\beta_{n}^{\left(m\right)}\right)$ and $\alpha^{\left(m\right)}$, for m=1,…,M, the above can be approximated using


$$\begin{array}{cc} f\left(y_{n+1}|y_{1},\ldots ,y_{n}\right) & \approx \frac{1}{M}\sum_{m=1}^{M}\left(\frac{\alpha^{\left(m\right)}}{\alpha^{\left(m\right)}+n}\right)\int f\left(y_{n+1}|\beta_{n+1}\right)g_{0}(\beta_{n+1})\;d\beta_{n+1}\\ & +\frac{1}{M}\sum_{m=1}^{M}\left(\frac{1}{\alpha^{\left(m\right)}+n}\sum_{j=1}^{n}f\left(y_{n+1}|\beta_{j}^{\left(m\right)}\right)\right). \end{array}$$ (6)

Next, we calculate the posterior predictive distribution for our BNP Poisson regression model in (3). On the right-hand side of Eq (6) above, *f* is the probability mass function of the Poisson distribution and *g*_0_ is the probability density function for the base probability distribution *G*_0_ which is the multivariate normal distribution with a (*k* + 1)-dimensional mean vector $0_{k+1}$ and a (*k* + 1) × (*k* + 1) covariance matrix *I*_*k*+1_ (i.e., the identity matrix of size *k* + 1). This means that


$$f\left(y_{n+1}|\beta_{n+1}\right)=\frac{e^{-t_{n+1}.e^{\left(x_{n+1}^{T}\beta_{n+1}\right)}}\times {\left(t_{n+1}.e^{\left(x_{n+1}^{T}\beta_{n+1}\right)}\right)}^{y_{n+1}}}{y_{n+1}!},$$

where *t*_*n*+1_ is a new exposure and $x_{n+1}$ is a new vector of covariates. For *g*_0_, we have


$$g_{0}(\beta_{n+1})=(2\pi {)}^{\frac{-(k+1)}{2}}e^{\frac{-1}{2}\beta_{n+1}^{T}\beta_{n+1}}.$$

We now split Eq (6) into two parts and calculate each part separately. First, we calculate the following integral:


$$\begin{array}{cc} \int f\left(y_{n+1}|\beta_{n+1}\right)g_{0}(\beta_{n+1})\;d\beta_{n+1} & =\int \frac{e^{-t_{n+1}.e^{\left(x_{n+1}^{T}\beta_{n+1}\right)}}\times {\left(t_{n+1}.e^{\left(x_{n+1}^{T}\beta_{n+1}\right)}\right)}^{y_{n+1}}}{y_{n+1}!}\\ & \times (2\pi {)}^{\frac{-(k+1)}{2}}e^{\frac{-1}{2}\beta_{n+1}^{T}\beta_{n+1}}\;d\beta_{n+1}. \end{array}$$

Let $h(\beta_{n+1})$ denote


$$h(\beta_{n+1})=-t_{n+1}.e^{\left(x_{n+1}^{T}\beta_{n+1}\right)}+y_{n+1}\left(\log(t_{n+1})+x_{n+1}^{T}\beta_{n+1}\right)-\frac{1}{2}\beta_{n+1}^{T}\beta_{n+1}.$$

Using multivariate Laplace approximation, we have


$$\begin{array}{cc} \int f\left(y_{n+1}|\beta_{n+1}\right)g_{0}(\beta_{n+1})\;d\beta_{n+1} & =\frac{(2\pi {)}^{\frac{-(k+1)}{2}}}{y_{n+1}!}\int \exp\left(h(\beta_{n+1})\right)\;d\beta_{n+1}\\ & \approx \frac{(2\pi {)}^{\frac{-(k+1)}{2}}}{y_{n+1}!}\exp\left(h({\hat{\beta }}_{n+1})\right)(2\pi {)}^{\frac{k+1}{2}}|\hat{\Sigma }{|}^{\frac{1}{2}}\\ & \approx \frac{1}{y_{n+1}!}\exp\left(h({\hat{\beta }}_{n+1})\right)|\hat{\Sigma }{|}^{\frac{1}{2}}, \end{array}$$

where ${\hat{\beta }}_{n+1}$ is the value of $\beta_{n+1}$ such that $\nabla h({\hat{\beta }}_{n+1})=0$ and $\hat{\Sigma }=[-\nabla^{2}h({\hat{\beta }}_{n+1}){]}^{-1}$ is the inverse of the negative Hessian of *h* evaluated at ${\hat{\beta }}_{n+1}$. Further details on the multivariate Laplace approximation are provided in the supporting information section (see S1 Appendix). Substituting this approximation into the expression for the posterior predictive distribution of *y*_*n*+1_ (Eq 6) yields


$$\begin{array}{ccc} & & f\left(y_{n+1}|y_{1},\ldots ,y_{n}\right)\approx \left(\frac{1}{M}\sum_{m=1}^{M}\frac{\alpha^{\left(m\right)}}{\alpha^{\left(m\right)}+n}\right)\frac{1}{y_{n+1}!}\exp\left(h({\hat{\beta }}_{n+1})\right){\left|\hat{\Sigma }\right|}^{\frac{1}{2}}\\ & & \;\;+\frac{1}{M}\sum_{m=1}^{M}\left(\frac{1}{\alpha^{\left(m\right)}+n}\sum_{j=1}^{n}\frac{1}{y_{n+1}!}\exp\left(-t_{n+1}.e^{\left(x_{n+1}^{T}\beta_{j}^{\left(m\right)}\right)}+y_{n+1}\left(\log(t_{n+1})+x_{n+1}^{T}\beta_{j}^{\left(m\right)}\right)\right)\right). \end{array}$$ (7)

Similarly, the posterior predictive distribution of our PYMM for claims frequency can be approximated using:


$$\begin{array}{ccc} & & f\left(y_{n+1}|y_{1},\ldots ,y_{n}\right)\approx \left(\frac{1}{M}\sum_{m=1}^{M}\frac{\alpha^{\left(m\right)}+d^{\left(m\right)}K_{n}^{\left(m\right)}}{\alpha^{\left(m\right)}+n}\right)\frac{1}{y_{n+1}!}\exp\left(h({\hat{\beta }}_{n+1})\right)|\hat{\Sigma }{|}^{\frac{1}{2}}\\ & & \;\;+\frac{1}{M}\sum_{m=1}^{M}\frac{1}{\left(\alpha^{\left(m\right)}+n\right)}(\sum_{j=1}^{n}\frac{1}{y_{n+1}!}\exp\left(-t_{n+1}.e^{\left(x_{n+1}^{T}\beta_{j}^{\left(m\right)}\right)}+y_{n+1}\left(\log(t_{n+1})+x_{n+1}^{T}\beta_{j}^{\left(m\right)}\right)\right)\\ & & \;\;-d^{\left(m\right)}\sum_{j=1}^{K_{n}^{\left(m\right)}}\frac{1}{y_{n+1}!}\exp\left(-t_{n+1}.e^{\left(x_{n+1}^{T}{\beta_{j}^{*}}^{\left(m\right)}\right)}+y_{n+1}\left(\log(t_{n+1})+x_{n+1}^{T}{\beta_{j}^{*}}^{\left(m\right)}\right)\right)). \end{array}$$ (8)

### 3.3 Posterior sampling

As discussed in Section 2, we use Neal’s Algorithm 8 to sample from the posterior distribution of the BNP regression parameters $\beta_{1},\ldots ,\beta_{n}$ presented in models (3) and (4). In the second step of Neal’s Algorithm 8, we draw a new value for the distinct parameter $\phi_{c}$ from the posterior distribution of $\left(\phi_{c}|y_{c}\right)$ when we assume that the parametric base probability measure *G*_0_ is the prior distribution (see [21]). Since the base probability measure *G*_0_ in our models (3) and (4) is a multivariate normal distribution and is not conjugate to Poisson likelihood, we replace the Gibbs sampling update for $\phi_{c}$ by a Metropolis–Hastings update to draw new values for $\phi_{c}$ at each MCMC iteration. The mean and variance of the multivariate normal proposal distribution are obtained using a Laplace approximation. As described in Section 2.1, for DPMM, we use the approach in [23] to update α at each MCMC iteration. For PYMM, we employ our method, detailed in Section 2.2 to update *d* and α during each MCMC iteration.

## 4 Results for insurance claim frequency

In this section, we evaluate the proposed BNP regression models for claim frequency using two independent real-world insurance datasets.

### 4.1 French motor insurance claims frequency dataset

The first dataset analyzed is the French motor insurance claims dataset, available as part of the R package CASdatasets. This data contains two datasets freMTPLfreq and freMTPLsev where risk features are collected for 413,169 motor third-part liability policies (observed mostly during a one-year period). In addition, we have claim numbers by policy as well as the corresponding claim amounts. The freMTPLfreq dataset contains the risk features and the claim number, while the freMTPLsev dataset contains the claim amount and the corresponding policy ID (see [31]). Here, we focus on the freMTPLfreq dataset, where claim numbers (*ClaimNb* column) serve as the response variable, and driver age (*DriverAge* column) and car age (*CarAge* column) are two covariates used in our BNP regression models:

- *PolicyID*: The policy ID (used to link with the claims dataset).
- *ClaimNb*: Number of claims during the exposure period.
- *Exposure*: The period of exposure for a policy, in years.
- *CarAge*: The vehicle age, in years.
- *DriverAge*: The driver age, in years (in France, people can drive a car at 18).

To assess the predictive capabilities of our BNP regression models, the data were randomly divided into a training set, used for model fitting, and a test set, used for predictive performance evaluation. We standardized each covariate to have mean zero and unit variance. With two covariates, our Dirichlet process mixture of Poisson regression model takes the form:


$$\begin{array}{cc} & y_{i}|\beta_{i}\;\overset{\mathrm{ind.}}{∼}\text{ Poisson}(t_{i}\exp(\beta_{i0}+\beta_{i1}x_{i1}+\beta_{i2}x_{i2}))\\ & \beta_{i}|G\;\overset{\mathrm{iid}}{∼}\;G\\ & G\;\sim \text{ DP}(\alpha ,G_{0})\\ & G_{0}\;=\text{ Multivariate-Normal}(0_{3},I_{3})\\ & \alpha \;\sim \text{ Gamma}(1,1). \end{array}$$

Similarly, our Pitman–Yor process mixture of Poisson regression model is:


$$\begin{array}{cc} & y_{i}|\beta_{i}\;\overset{\mathrm{ind.}}{∼}\text{ Poisson}(t_{i}\exp(\beta_{i0}+\beta_{i1}x_{i1}+\beta_{i2}x_{i2}))\\ & \beta_{i}|G\;\overset{\mathrm{iid}}{∼}\;G\\ & G\;\sim \text{ PY}(d,\alpha ,G_{0})\\ & G_{0}\;=\text{ Multivariate-Normal}(0_{3},I_{3})\\ & d\;\sim \text{ Uniform}(0,1)\\ & (\alpha +d)|d\;\sim \text{ Log-Normal}(0,1). \end{array}$$

The MCMC sampling algorithm was implemented in R and run for 50,000 iterations, with the initial 25,000 iterations discarded as burn-in, on the DEAC high-performance computing cluster at Wake Forest University [32]. The total computational times was approximately 23.4 hours for the DPMM and 23.6 hours for the PYMM when running on a single core of one node. These durations include both MCMC sampling and computation of the posterior predictive distributions. Markov chain mixing and convergence to the stationary distribution were assessed using trace plots, autocorrelation function plots, and diagnostic tests from the R package coda (see [33]); all indicators suggested good convergence.

**Fig 1:**
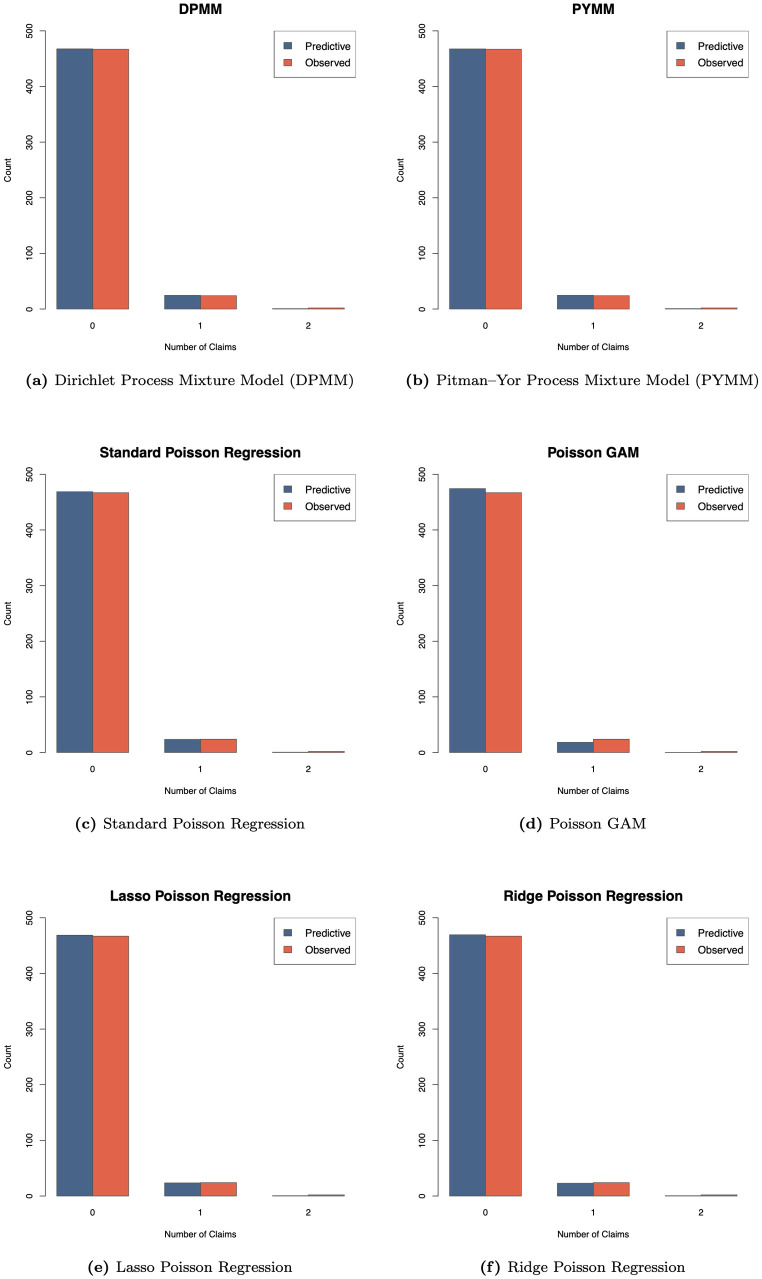
Comparison of predictive distributions and observed distribution of the number of claims across six models for the French insurance dataset. Each panel displays the model’s predictive distribution (blue bars) and the observed distribution of claim numbers (red bars) for a test subgroup with *car age* = 2 and *driver age* = 62 in the French motor claims frequency dataset. The title within each panel identifies the specific model used.

For illustrative purposes, we computed the posterior predictive distributions of the future number of claims for a class of insurance policies with car age 2 and driver age 62 (observed during a one-year period), as described in Eq (7) and (8). Fig 1 compares the model-predicted and observed claim number distributions for six fitted models: our two BNP regression models (DPMM and PYMM) and four alternative regression approaches, including the standard parametric Poisson regression and the semi-parametric or penalized regression methods (Lasso, Ridge, and Poisson GAM). The plots indicate that the BNP models (DPMM and PYMM) more accurately capture the empirical distribution of the test data, whereas the classical, semi-parametric, and penalized models show less flexibility in reproducing the observed distribution of claim numbers.

To evaluate the predictive performance of the proposed BNP models (DPMM and PYMM) relative to alternative approaches, we computed the mean squared error (MSE) of predicted distributions with respect to holdout test data. This metric, described in the note to Table 1, quantifies the average squared difference between the model-predicted and observed distributions of number of claims for the selected test subgroup. Table 1 reports the MSE values along with their corresponding standard errors (SEs), rounded to four decimal places. The BNP models, PYMM (MSE = 0.7856) and DPMM (MSE = 0.8283), show the best predictive performance, substantially outperforming all other models. In contrast, the standard parametric Poisson regression exhibits a higher MSE (1.7223), while the penalized and semi-parametric models (Lasso, Ridge, and GAM) show progressively larger MSEs. Overall, the BNP models demonstrate superior predictive accuracy and greater stability compared to both the classical and penalized regression alternatives.

**Table 1:** Model comparison based on mean squared error (MSE) of predicted distributions and corresponding standard errors (SE) for the French motor claims frequency dataset.

Model	MSE	SE of MSE
Pitman–Yor Process Mixture Model (PYMM)	0.7856	0.4335
Dirichlet Process Mixture Model (DPMM)	0.8283	0.4391
Standard Poisson Regression	1.7223	0.8578
Lasso Poisson Regression	1.7486	0.8708
Ridge Poisson Regression	3.1002	1.5427
Poisson GAM	29.2024	14.5941

**Note.** The MSE of predicted distributions represents the mean squared error between the model-predicted and observed distributions of number of claims for the test subgroup (*car age* = 2, *driver age* = 62).

To quantify the predictive gains of the proposed BNP models relative to the standard parametric Poisson regression (baseline), we computed the percentage improvement in predictive performance using the following formula for percentage reduction in MSE:


$$\text{Percentage Improvement}=\left(\frac{{\text{MSE}}_{\text{base}}-{\text{MSE}}_{\text{proposed}}}{{\text{MSE}}_{\text{base}}}\right)\times 100\%$$ (9)

where MSE_base_ denotes the MSE of the standard parametric model and MSE_proposed_ corresponds to that of the BNP model being evaluated. Using the MSE values derived for the test subgroup, both BNP models demonstrated substantial improvements. The PYMM reduced the MSE from 1.7223 (standard Poisson regression) to 0.7856, resulting in a 54.4% improvement in predictive accuracy, while the DPMM reduced the MSE to 0.8283, achieving a 51.9% improvement. These results indicate that both BNP models lower the MSE by roughly 52% relative to the classical parametric Poisson model, highlighting their superior predictive performance for the French motor claims frequency data.

**Fig 2:**
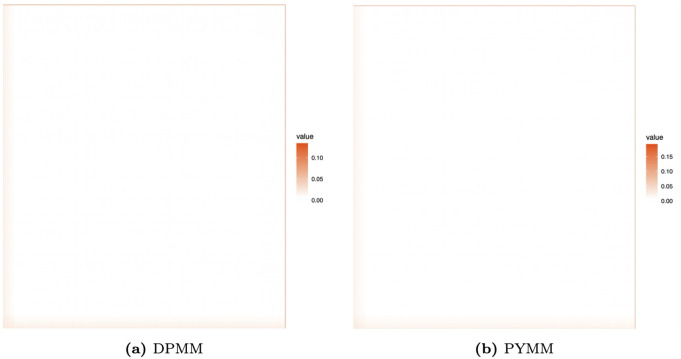
Heat maps of the dissimilarity matrices showing clustering structure for the French motor claims frequency dataset.

We also assessed the posterior clustering performance of our BNP regression models for the French motor insurance claims frequency data. Clustering was performed based on samples from the posterior distribution of $\beta_{1},\ldots ,\beta_{n}$. As detailed in Section 2.3, we constructed the *n* × *n* dissimilarity matrix from these posterior samples. Heat maps of the dissimilarity matrix for this data are presented in Fig 2. In these visualizations, observations are arranged along both the horizontal and vertical axes, where light-colored blocks signify identified clusters. From these maps, we observe approximately one cluster, suggesting that the French motor insurance claims frequencies could possibly be modeled using a single parametric Poisson regression.

From an actuarial perspective, this finding suggests that, once car age and driver age are taken into account, the portfolio is relatively homogeneous with respect to claim frequency: most policies exhibit similar underlying claim rates, and there is no strong evidence of clearly separated groups with very different claim frequencies. In practice, this suggests that a single Poisson regression model may be sufficient for modeling the average number of claims using these covariates alone. At the same time, the BNP models still deliver clear gains in predictive performance (Table 1), indicating that their main advantage for frequency lies in flexibly capturing overdispersion and local deviations from the Poisson assumption, rather than in uncovering well-separated clusters. In other words, while the cluster structure does not reveal clearly separated high- and low-frequency groups, the BNP approach provides a more realistic description of the distribution of the number of claims and thus more accurate predictions for pricing and risk management.

### 4.2 Belgian motor insurance claims frequency dataset

The second dataset we analyze is a Belgian motor third-part liability dataset (beMTPL97), available in the R package CASdatasets. The portfolio contains 163,212 motor third-part liability policies observed during the year 1997 (see [31]). Unlike the French data, this dataset combines risk features and claim information in a single structure. Claim information is available in terms of both the number of claims and the average claim amount (in euros) reported during the exposure period. Here, we focus on modeling claim frequency, where the claim number (*nclaims* column) serves as the response variable. The dataset also includes a rich set of policyholder and vehicle risk characteristics, including policyholder age (*ageph* column) and vehicle age in years (*agec* column), which we use as covariates in our BNP regression models. In addition to claim frequency, the dataset provides claim severity information through the average claim amount (*average* column). The main variables used in our claim frequency and severity analysis are:

- *id*: a numeric for the policy number.
- *nclaims*: a numeric for the claim number.
- *average*: a numeric for the average claim amount.
- *expo*: a numeric for the exposure.
- *agec*: a numeric for age of the vehicle in years.
- *ageph*: a numeric for the policyholder age.

**Fig 3:**
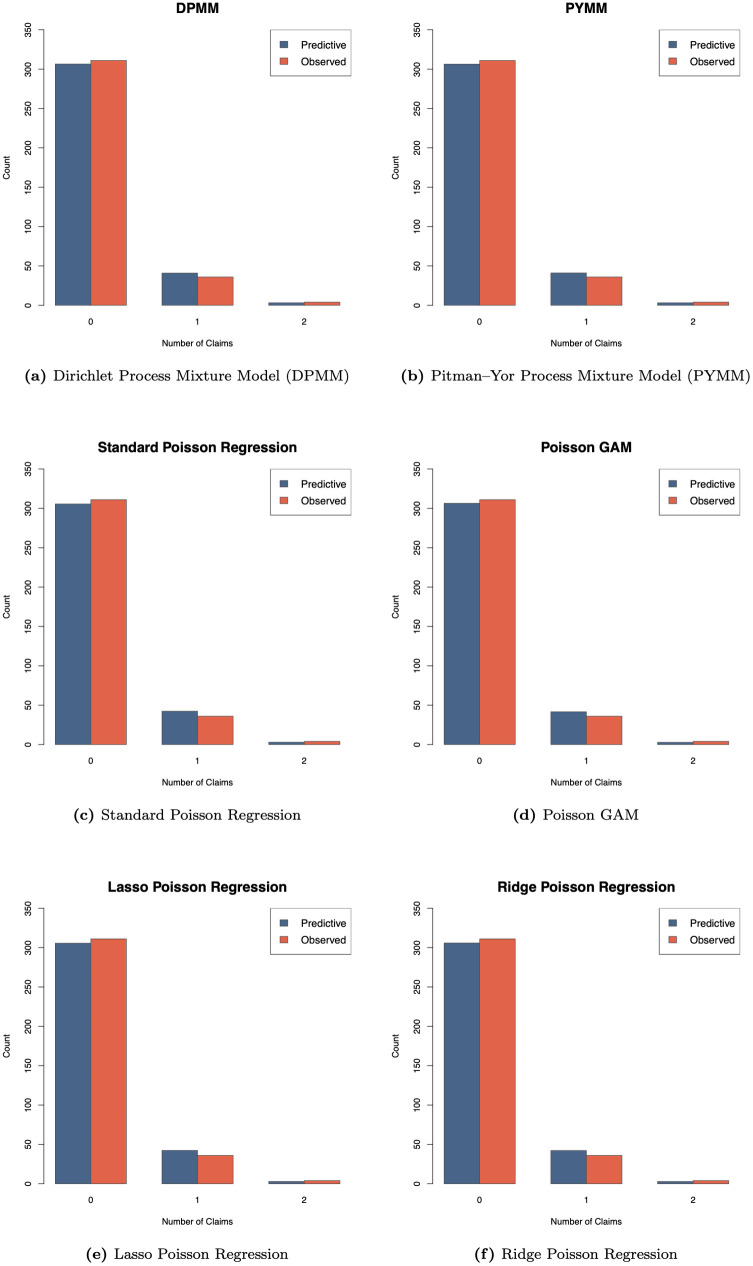
Comparison of predictive and observed claim number distributions across six models for the Belgian insurance dataset. Each panel displays the model’s predictive distribution (blue bars) and the observed distribution of claim numbers (red bars) for a test subgroup with *car age* = 6 and *driver age* = 39 in the Belgian motor claims frequency dataset. The title within each panel identifies the specific model used.

We randomly partitioned the data into training and test sets and standardized each covariate to have zero mean and unit variance. The MCMC sampling was run for 50,000 iterations, discarding the first 25,000 as burn-in. Computation took approximately 17.5 hours for the DPMM and 17.9 hours for the PYMM using a single core on one node of the DEAC high-performance computing cluster at Wake Forest University [32]. As a representative example, Fig 3 shows how the six fitted models reproduce the claim frequency distribution for Belgian motor insurance policies with car age 6 and driver age 39, by comparing each model’s predictive distribution with the empirical distribution observed in the test data.

Table 2 summarizes predictive performance for the Belgian dataset using the MSE metric for the selected test subgroup. Consistent with the French analysis, the two BNP models again perform best, yielding the lowest MSE values among all fitted models. The DPMM achieved the best performance with an MSE of 15.1088, followed by the PYMM at 16.0687, whereas the standard Poisson regression produced a substantially higher MSE of 23.6712. The penalized and semi-parametric models (Lasso, Ridge, and GAM) also failed to match the precision of the BNP models. Relative to the standard Poisson baseline, the DPMM and PYMM reduced the MSE by approximately 36.2% and 32.1%, respectively, indicating meaningful gains in predictive performance for the Belgian motor claims frequency data.

**Table 2:** Model comparison based on mean squared error (MSE) of predicted distributions and corresponding standard errors (SE) for the Belgian motor claims frequency dataset.

Model	MSE	SE of MSE
Pitman–Yor Process Mixture Model (PYMM)	16.0687	7.8109
Dirichlet Process Mixture Model (DPMM)	15.1088	7.3475
Standard Poisson Regression	23.6712	11.6993
Lasso Poisson Regression	23.7281	11.7278
Ridge Poisson Regression	22.1553	10.9400
Poisson GAM	17.9813	8.8500

**Note.** The MSE of predicted distributions represents the mean squared error between the model-predicted and observed distributions of number of claims for the test subgroup (*car age* = 6, *driver age* = 39).

**Fig 4:**
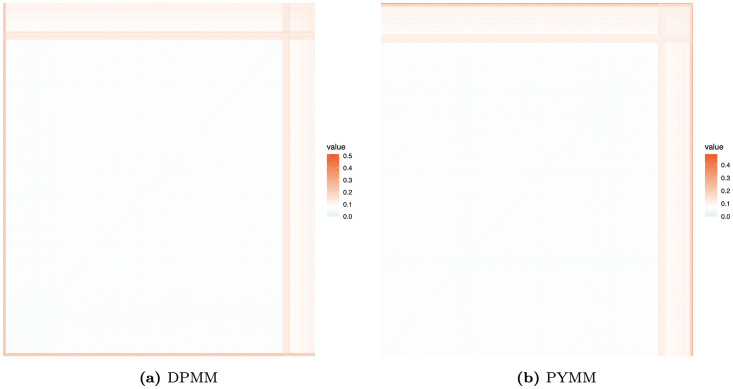
Heat maps of the dissimilarity matrices showing clustering structure for the Belgian motor claims frequency dataset.

Fig 4 displays the posterior clustering heat maps for the Belgian motor claims frequency data. The heat maps suggest the presence of approximately two clusters, indicating heterogeneity in claim frequency. This clustering structure implies that a single Poisson regression is likely insufficient for this dataset, highlighting the added flexibility of the proposed BNP models.

## 5 Modeling claims severity

The second step in developing an insurance pricing model involves predicting claims severity, which is the amount of each claim. For *n* insurance policies, each with a set of *k* covariates, we denote the *i*th policy’s claim amount as *z*_*i*_ and the *i*th covariate vector as $x_{i}=(1,x_{i1},\ldots ,x_{ik}{)}^{T}$. The parametric normal regression (multiple linear regression) model, widely used to model the log(claim amounts), is:


$$\log(z_{i})|(\beta ,\sigma^{2})\;\overset{\mathrm{ind.}}{∼}\text{ Normal}(x_{i}^{T}\beta ,\sigma^{2})\;;\;i=1,\ldots ,n.$$

However, this model assumes that the response to covariates is similar across individuals, as each data point shares the same regression parameter vector $\left(\beta ,\sigma^{2}\right)$, where β represents the regression coefficients and $\sigma^{2}$ is the error variance. Alternatively, DPMM and PYMM allow each data point *i* to have its own regression parameter vector, $\left(\beta_{i},\sigma_{i}^{2}\right)$, and due to the clustering property of the DP and PY priors, data points are then clustered by their shared regression parameters.

### 5.1 Model description

Here, the parameters of the BNP regression model are $\theta_{i}=(\beta_{i},\sigma_{i}^{2})$, for each individual i=1,…,n, where $\beta_{i}=(\beta_{i0},\beta_{i1},\ldots ,\beta_{ik})$ is the vector of regression coefficients and $\sigma_{i}^{2}$ is the error variance. We only consider policies with positive claim amounts and model these amounts on the log scale. We propose two BNP models for the log(claim amounts) data as follows.

First, we define a DP prior on the mixing distribution over the regression parameters, $\theta_{i}=(\beta_{i},\sigma_{i}^{2})$, and model the logarithm of claim amounts using a Dirichlet process mixture of normal regression:


$$\begin{array}{cc} & \log(z_{i})|(\beta_{i},\sigma_{i}^{2})\;\overset{\mathrm{ind.}}{∼}\text{ Normal}(x_{i}^{T}\beta_{i},\sigma_{i}^{2})\;;\;i=1,\ldots ,n\\ & \theta_{i}=(\beta_{i},\sigma_{i}^{2})|G\;\overset{\mathrm{iid}}{∼}\;G\;;\;i=1,\ldots ,n\\ & G\;\sim \text{ DP}(\alpha ,G_{0})\\ & G_{0}\;=\;G_{0_{\beta_{i}|\sigma_{i}^{2}}}\times G_{0_{\sigma_{i}^{2}}}\\ & G_{0_{\beta_{i}|\sigma_{i}^{2}}}\;=\text{ Multivariate-Normal}(0_{k+1},n_{0}\sigma_{i}^{2}I_{k+1})\\ & G_{0_{\sigma_{i}^{2}}}\;=\text{ Inverse-Gamma}(a,b)\\ & \alpha \;\sim \text{ Gamma}(1,1). \end{array}$$ (10)

The coefficient *n*_0_ plays a crucial role in the covariance matrix of the multivariate normal distribution and must be carefully specified. We determined an effective value for this through trial and error in practice, finding that *n*_0_ = 0.5 works well here. $G_{0_{\sigma_{i}^{2}}}$ is the base distribution for $\sigma_{i}^{2}$ which is chosen to be an inverse gamma distribution with shape parameter *a* = 3 and scale parameter *b* = 5.

Alternatively, we can set a PY prior instead of a DP prior and model the logarithm of claim amounts using a Pitman–Yor process mixture of normal regression:


$$\begin{array}{cc} & \log(z_{i})|(\beta_{i},\sigma_{i}^{2})\;\overset{\mathrm{ind.}}{∼}\text{ Normal}(x_{i}^{T}\beta_{i},\sigma_{i}^{2})\;;\;i=1,\ldots ,n\\ & \theta_{i}=(\beta_{i},\sigma_{i}^{2})|G\;\overset{\mathrm{iid}}{∼}\;G\;;\;i=1,\ldots ,n\\ & G\;\sim \text{ PY}(d,\alpha ,G_{0})\\ & G_{0}\;=\;G_{0_{\beta_{i}|\sigma_{i}^{2}}}\times G_{0_{\sigma_{i}^{2}}}\\ & G_{0_{\beta_{i}|\sigma_{i}^{2}}}\;=\text{ Multivariate-Normal}(0_{k+1},n_{0}\sigma_{i}^{2}I_{k+1})\\ & G_{0_{\sigma_{i}^{2}}}\;=\text{ Inverse-Gamma}(a,b)\\ & d\sim \text{Uniform}(0,1)\\ & (\alpha +d)|d\;\sim \text{ Log-Normal}(0,1). \end{array}$$ (11)

### 5.2 Computation of posterior predictive distributions

Henceforth, we use $y_{i}=\log(z_{i})$ to denote the log(claim severity) for the *i*th individual. We are interested in the posterior predictive distribution for the future log(claim severity), *y*_*n*+1_. Following similar steps as in the computation of the posterior predictive distribution of DPMM for claims frequency in Section 3.2 and Eq 6, we have


$$\begin{array}{cc} f\left(y_{n+1}|y_{1},\ldots ,y_{n}\right) & \approx \frac{1}{M}\sum_{m=1}^{M}\left(\frac{\alpha^{\left(m\right)}}{\alpha^{\left(m\right)}+n}\right)\int f\left(y_{n+1}|\theta_{n+1}\right)g_{0}(\beta_{n+1})\;d\theta_{n+1}\\ & +\frac{1}{M}\sum_{m=1}^{M}\left(\frac{1}{\alpha^{\left(m\right)}+n}\sum_{j=1}^{n}f\left(y_{n+1}|\theta_{j}^{\left(m\right)}\right)\right). \end{array}$$

Since the base measure *G*_0_ in the model (10) is a conjugate prior to the likelihood given by this model, the integral $\int f\left(y_{n+1}|\theta_{n+1}\right)g_{0}(\beta_{n+1})\;d\theta_{n+1}$ above can be calculated in closed form. Following similar steps in Section 3.2, it can be shown that the posterior predictive distribution of the log(claims severity) for DPMM is equal to:


$$\begin{array}{cc} f\left(y_{n+1}|y_{1},\ldots ,y_{n}\right) & \approx \frac{1}{M}\sum_{t=1}^{M}\left(\frac{\alpha^{\left(m\right)}}{\alpha^{\left(m\right)}+n}\right)\\ & \times \left(\frac{1}{\sqrt{2\pi }}\frac{1}{n_{0}^{\frac{k+1}{2}}}\frac{b^{a}}{\Gamma (a)}\frac{1}{\sqrt{det(U)}}\frac{\Gamma (\frac{1}{2}+a)}{{\left[b+\frac{1}{2}y_{n+1}^{2}-\frac{1}{2}d^{T}U^{-1}d\right]}^{\left(\frac{1}{2}+a\right)}}\right)\\ & +\frac{1}{M}\sum_{t=1}^{M}\left(\frac{1}{\alpha^{\left(m\right)}+n}\sum_{j=1}^{n}f\left(y_{n+1}|\theta_{j}^{\left(m\right)}\right)\right), \end{array}$$ (12)

where $U=x_{n+1}x_{n+1}^{T}+\frac{1}{n_{0}}I$ and $d^{T}=y_{n+1}x_{n+1}^{T}$.

Similarly, the posterior predictive distribution of the log(claims severity) for PYMM equals:


$$\begin{array}{cc} f\left(y_{n+1}|y_{1},\ldots ,y_{n}\right) & \approx \left(\frac{1}{M}\sum_{t=1}^{M}\frac{\alpha^{\left(m\right)}+d^{\left(m\right)}K_{n}^{\left(m\right)}}{\alpha^{\left(m\right)}+n}\right)\\ & \times \left(\frac{1}{\sqrt{2\pi }}\frac{1}{n_{0}^{\frac{k+1}{2}}}\frac{b^{a}}{\Gamma (a)}\frac{1}{\sqrt{det(U)}}\frac{\Gamma (\frac{1}{2}+a)}{(b+\frac{1}{2}y_{n+1}^{2}-\frac{1}{2}d^{T}U^{-1}d{)}^{\left(\frac{1}{2}+a\right)}}\right)\\ & +\frac{1}{M}\sum_{t=1}^{M}\frac{1}{\left(\alpha^{\left(m\right)}+n\right)}\left(\sum_{j=1}^{n}f\left(y_{n+1}|\theta_{j}^{\left(m\right)}\right)-d^{\left(m\right)}\sum_{j=1}^{K_{n}^{\left(m\right)}}f\left(y_{n+1}|{\theta_{j}^{*}}^{\left(m\right)}\right)\right), \end{array}$$ (13)

where $U=x_{n+1}x_{n+1}^{T}+\frac{1}{n_{0}}I$ and $d^{T}=y_{n+1}x_{n+1}^{T}$.

### 5.3 Posterior sampling

As discussed in Section 2, Neal’s Algorithm 8 can also be employed for models with conjugate priors to avoid computing the integrals $\int g_{0}(\theta )f(y_{i}|\theta )\;d\theta$ for the conditional probabilities within the MCMC algorithm. In the second step of this algorithm, we draw a new value for the distinct parameter $\phi_{c}$ from the posterior distribution of $\left(\phi_{c}|y_{c}\right)$ when we assume that the parametric base probability distribution *G*_0_ is the prior distribution (see [21]). Since the probability base distribution *G*_0_ in models (10) and (11) is a conjugate prior to the likelihood of the models, we use this conjugacy to implement the Gibbs sampling update where a new value for $\phi_{c}$ is drawn from its posterior distribution given the data associated with the cluster label *c*.

## 6 Results for insurance claim severity

In this section, we apply the proposed BNP regression models to analyze claim severity, again using two independent real-world insurance datasets.

### 6.1 French motor insurance claims severity dataset

First, we focus on the freMTPLsev dataset, which contains nonzero claim amounts for 16,181 motor third-part liability policies (observed mostly during a one-year period). This dataset has 2 columns as follows:

- *PolicyID*: The policy ID (used to link with the contract dataset).
- *ClaimAmount*: The cost of the claim, seen as at a recent date.

First, we linked the freMTPLsev dataset with the freMTPLfreq dataset using the PolicyID column to incorporate risk features. We then used the log-transformed claim amounts (*ClaimAmount* column) as the response variable and included driver age (*DriverAge* column) and car age (*CarAge* column) as two covariates in our BNP regression models. Data were randomly partitioned into training and testing. With two covariates, our Dirichlet process mixture of normal regression model is:


$$\begin{array}{cc} & y_{i}|(\beta_{i},\sigma_{i}^{2})\;\overset{\mathrm{ind.}}{∼}\text{ Normal}(\beta_{i0}+\beta_{i1}x_{i1}+\beta_{i2}x_{i2},\sigma_{i}^{2})\\ & \theta_{i}=(\beta_{i},\sigma_{i}^{2})|G\;\overset{\mathrm{iid}}{∼}\;G\\ & G\;\sim \text{ DP}(\alpha ,G_{0})\\ & G_{0}\;=\;G_{0_{\beta_{i}|\sigma_{i}^{2}}}\times G_{0_{\sigma_{i}^{2}}}\\ & G_{0_{\beta_{i}|\sigma_{i}^{2}}}\;=\text{ Multivariate-Normal}(0_{3},0.5\sigma_{i}^{2}I_{3})\\ & G_{0_{\sigma_{i}^{2}}}\;=\text{ Inverse-Gamma}(3,5)\\ & \alpha \;\sim \text{ Gamma}(1,1). \end{array}$$

Similarly, our Pitman–Yor process mixture of normal regression model is:


$$\begin{array}{cc} & y_{i}|(\beta_{i},\sigma_{i}^{2})\;\overset{\mathrm{ind.}}{∼}\text{ Normal}(\beta_{i0}+\beta_{i1}x_{i1}+\beta_{i2}x_{i2},\sigma_{i}^{2})\\ & \theta_{i}=(\beta_{i},\sigma_{i}^{2})|G\;\overset{\mathrm{iid}}{∼}\;G\\ & G\;\sim \text{ PY}(d,\alpha ,G_{0})\\ & G_{0}\;=\;G_{0_{\beta_{i}|\sigma_{i}^{2}}}\times G_{0_{\sigma_{i}^{2}}}\\ & G_{0_{\beta_{i}|\sigma_{i}^{2}}}\;=\text{ Multivariate-Normal}(0_{3},0.5\sigma_{i}^{2}I_{3})\\ & G_{0_{\sigma_{i}^{2}}}\;=\text{ Inverse-Gamma}(3,5)\\ & d\sim \text{Uniform}(0,1)\\ & (\alpha +d)|d\;\sim \text{ Log-Normal}(0,1). \end{array}$$

For the claims severity analysis, the MCMC sampling algorithm was implemented in R and run for 50,000 iterations, discarding the initial 25,000 iterations as burn-in. Computations were performed on the DEAC high-performance computing cluster at Wake Forest University [32]. The total runtime was approximately 26.3 hours for the DPMM and 26.4 hours for the PYMM when running on a single core of one node. These durations include both MCMC sampling and computation of the posterior predictive distributions. Markov chain convergence was confirmed by trace plots, autocorrelation function plots, and diagnostic tests from the the R package coda, none of which indicated any issues with convergence.

**Fig 5:**
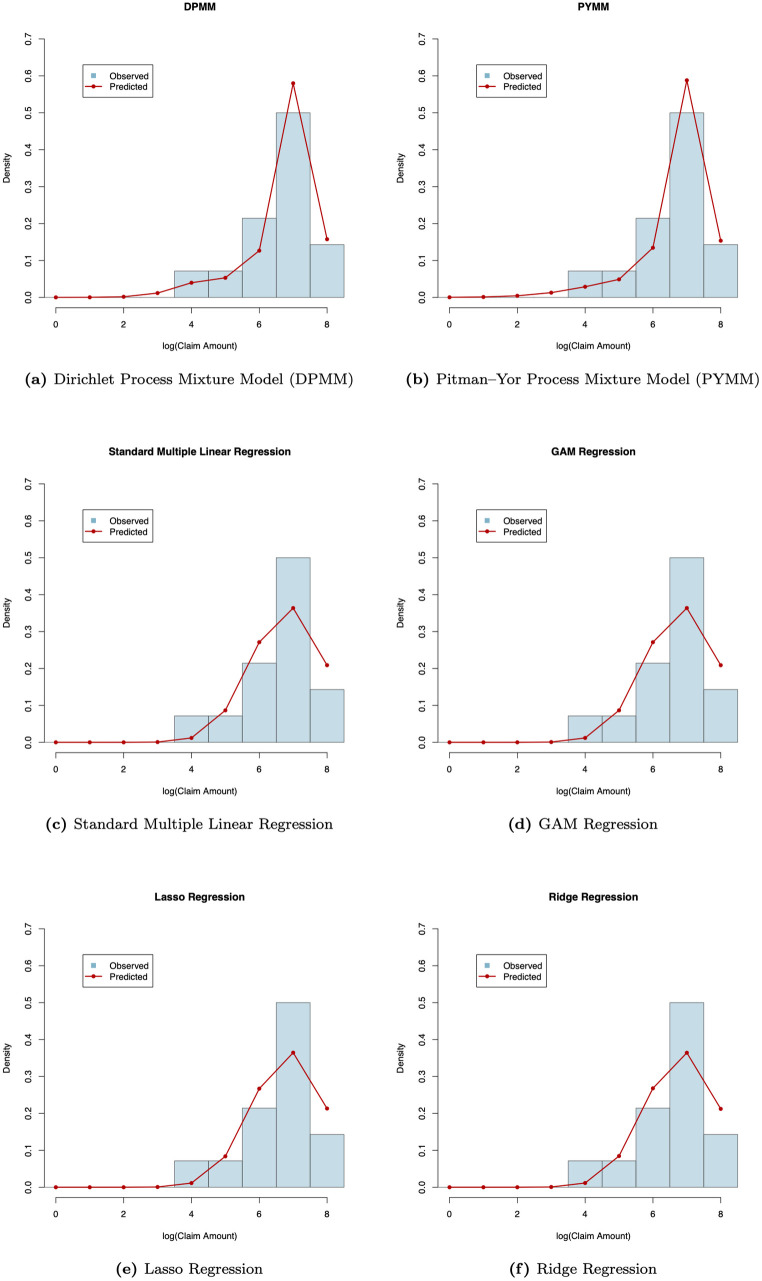
Comparison of predictive and observed distributions of log(claim amounts) across six models for the French insurance dataset. Each panel displays the model’s predictive density (red line) and the observed distribution of log(claim amounts) (blue histogram) for a test subgroup with *car age* = 9 and *driver age* = 38 in the French motor claims severity dataset. Each panel title indicates the corresponding model.

For illustration, we computed the posterior predictive densities of the future log(claim amounts) for a representative class of insurance policies with car age 9 and driver age 38, following Eq (12) and (13). Fig 5 presents a comparison between the predicted and observed distributions obtained from six fitted models: the two proposed BNP regression models (DPMM and PYMM) and four alternative regression approaches, including the standard multiple linear regression, Lasso regression, Ridge regression, and GAM regression. As shown in the figure, the BNP models (DPMM and PYMM) more accurately capture the empirical distribution of the test data, while the alternative models show noticeably poorer fit and less flexibility in capturing the observed distribution of log(claim amounts).

To assess the predictive performance of the proposed BNP models (DPMM and PYMM) in comparison with alternative regression approaches, we calculated the mean squared error (MSE) of the predicted distributions using the holdout test data. This metric, defined in the note to Table 3, measures the average squared difference between the model-predicted and observed distributions of log(claim amounts) for the selected test subgroup. Table 3 summarizes the MSE values and their corresponding standard errors (SEs). Consistent with the claims frequency analysis, the BNP models achieve the lowest MSEs, 0.3436 for DPMM and 0.3649 for PYMM, substantially outperforming all alternative models. The remaining approaches, including the standard multiple linear, Lasso, Ridge, and GAM regressions, yield notably higher MSEs, confirming the superior predictive accuracy and stability of the BNP models for the claims severity data.

**Table 3:** Model comparison based on mean squared error (MSE) of predicted distributions and corresponding standard errors (SE) for the French motor claims severity dataset.

Model	MSE	SE of MSE
Dirichlet Process Mixture Model (DPMM)	0.3436	0.1980
Pitman–Yor Process Mixture Model (PYMM)	0.3649	0.1975
Standard Multiple Linear Regression	0.6524	0.3917
Lasso Regression	0.6501	0.3885
Ridge Regression	0.6510	0.3898
GAM Regression	0.6524	0.3917

**Note.** The MSE of predicted distributions represents the mean squared error between the model-predicted and observed distributions of log(claim amounts) for the test subgroup (*car age* = 9, *driver age* = 38).

To further quantify the predictive improvements achieved by the BNP models in the claims severity analysis, we used the percentage reduction in MSE defined in Eq (9), taking the standard multiple linear regression model as the baseline. Based on the MSE values in Table 3, the DPMM reduced the MSE from 0.6524 (baseline) to 0.3436, corresponding to a 47.3% improvement in predictive accuracy, while the PYMM achieved a comparable 44.1% improvement with an MSE of 0.3649. These results indicate that both BNP models lower the prediction error by nearly 45% relative to the classical parametric regression, reinforcing their superior performance for modeling log(claim amounts) in the French motor claims severity dataset.

**Fig 6:**
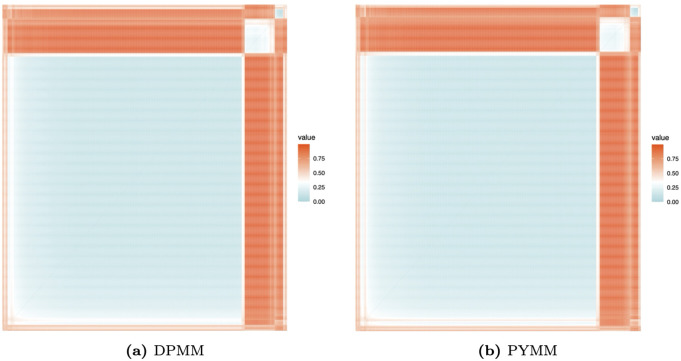
Heat maps of the dissimilarity matrices showing clustering structure for the French motor claims severity dataset.

**Fig 7:**
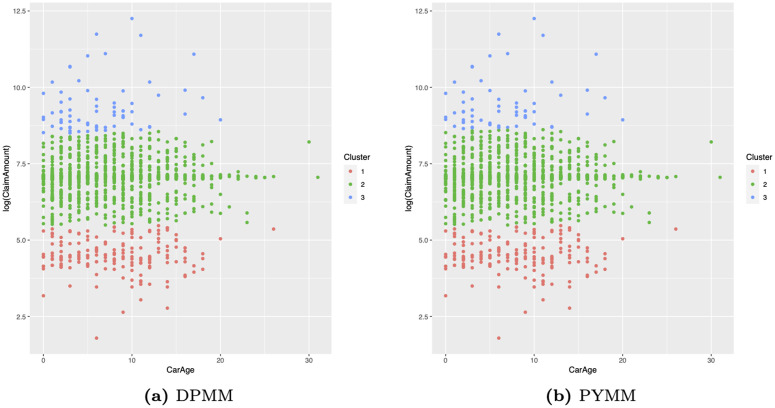
Scatter plots of log(claim amounts) versus car age for the French motor claims severity dataset.

**Fig 8:**
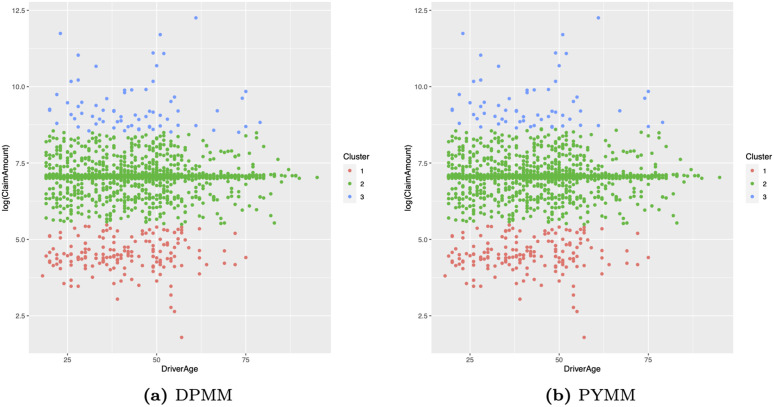
Scatter plots of log(claim amounts) versus driver age for the French motor claims severity dataset.

Fig 6 presents heat maps of the dissimilarity matrix for the French motor insurance claims severity data, derived from the two BNP models. In these maps, light-colored (blue or white) blocks along the diagonal indicate clusters. The heat maps reveal approximately three clusters with distinct areas. This suggests that the French motor insurance claims severity data on the log scale could be modeled using a mixture of three normal regression models with varying weights. We also illustrate the clustering structure of our BNP regression models in scatter plots of log-transformed claim amounts versus car age (Fig 7) and driver age (Fig 8). The first cluster (in red) includes observations with log(claim amounts) approximately less than 5.25, the second cluster (in green) covers log(claim amounts) between approximately 5.25 and 8.50, and the third cluster (in blue) consists of observations with log(claim amounts) approximately greater than 8.50.

From an insurance portfolio perspective, these three clusters can be interpreted as latent severity levels: low-cost claims, medium-sized claims, and large claims. Importantly, these latent clusters are learned automatically by the BNP regression models, without pre-specifying any thresholds or bands for log(claim amounts), providing a data-driven view of how log(claim amounts) naturally group in this portfolio. Moreover, the fact that these clusters emerge when conditioning on car age and driver age suggests that, even with a limited set of covariates, the BNP models can uncover meaningful heterogeneity in severity profiles that would not be visible under a single normal regression model.

Figs 7 and 8 further relate these clusters to observable risk characteristics. In the scatter plots of log(claim amounts) versus car age and driver age, the three clusters overlap in the covariate space, indicating that extremely large claims cannot be explained solely by simple linear effects of car age or driver age. This highlights the presence of unobserved heterogeneity in claim severity that is not captured by these covariates alone. Actuarially, this has important implications: the large-claims cluster is precisely the portion of the portfolio that is most important for reinsurance design and capital allocation, whereas the lower two clusters are mainly relevant for everyday pricing decisions. A practical advantage is that an insurer could use the posterior cluster allocations to identify policies belonging to the large-claims cluster and then tailor underwriting rules and deductibles for those risks. Thus, the clustering output is not only a by-product of the BNP approach but also a tool for understanding and managing severity risk within the portfolio.

### 6.2 Belgian motor insurance claims severity dataset

**Fig 9:**
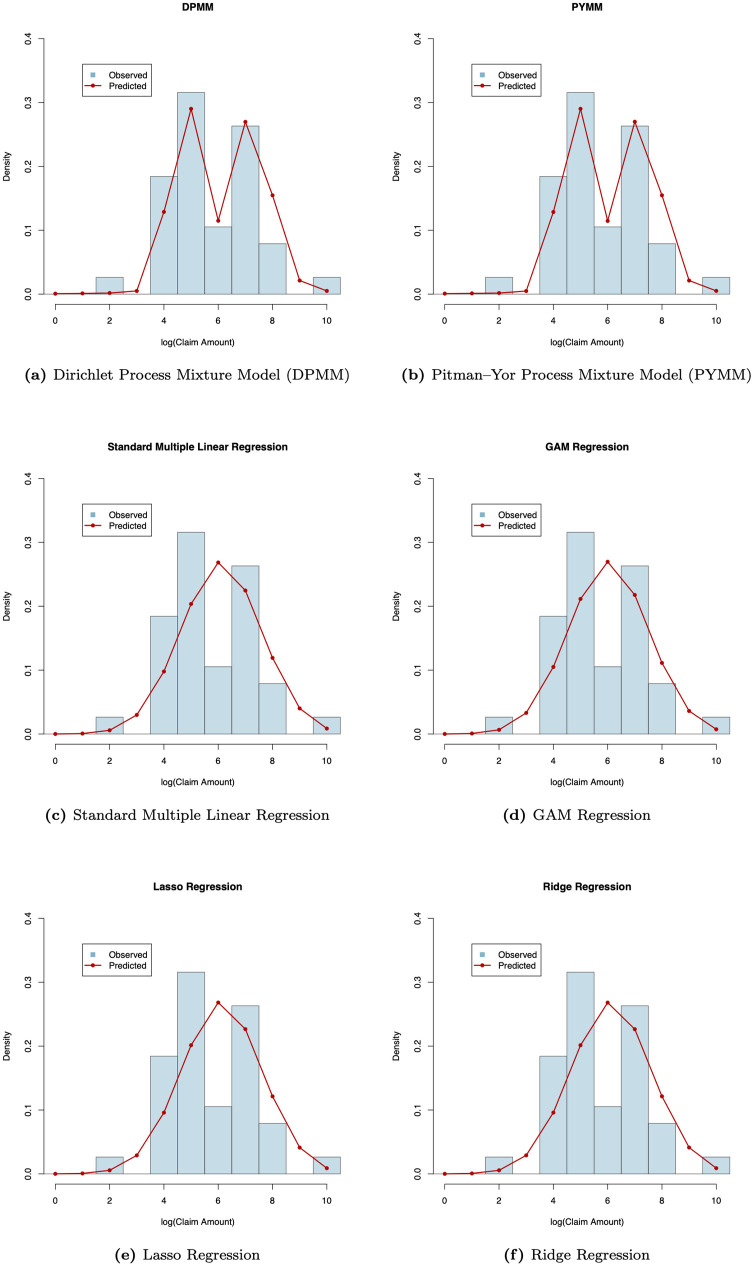
Comparison of predictive and observed distributions of log(claim amounts) across six models for the Belgian insurance dataset. Each panel displays the model’s predictive density (red line) and the observed distribution of log(claim amounts) (blue histogram) for a test subgroup with *car age* = 6 and *driver age* = 39 in the Belgian motor claims severity dataset. Each panel title indicates the corresponding model.

Second, we analyze the Belgian motor third-part liability dataset (beMTPL97). In this analysis, we focus on modeling claim severity, where the log-transformed nonzero claim amount serves as the response variable, while policyholder age and vehicle age in years are used as the covariates in our BNP regression models. Data were randomly divided into training and testing sets. We performed MCMC sampling for 50,000 iterations, treating the initial 25,000 as burn-in. Computations were carried out on a single core of the DEAC high-performance computing cluster at Wake Forest University [32] and required approximately 24.5 hours for each of the DPMM and PYMM models. As an illustrative case, Fig 9 compares the model-predicted and observed distributions of log-transformed claim severity for Belgian motor insurance policies with car age 6 and driver age 39 across the six fitted models.

Table 4 presents the MSE (and SE) of the predicted distributions for log-transformed nonzero claim amounts on the Belgian dataset for the selected test subgroup. Consistent with the results from the French severity analysis, the BNP models provide the most accurate predictions, with the DPMM and PYMM yielding the lowest errors (MSE = 1.4629 and 1.4662, respectively), while the regression alternatives have substantially larger MSEs (e.g., 6.9634 for standard multiple linear regression). Using the standard multiple linear regression as the baseline, the DPMM and PYMM achieved approximately 79.0% and 78.9% reductions in MSE, respectively, confirming the predictive advantage of the proposed BNP models for modeling claim severity in the Belgian motor insurance dataset.

**Table 4:** Model comparison based on mean squared error (MSE) of predicted distributions and corresponding standard errors (SE) for the Belgian motor claims severity dataset.

Model	MSE	SE of MSE
Dirichlet Process Mixture Model (DPMM)	1.4629	0.7826
Pitman–Yor Process Mixture Model (PYMM)	1.4662	0.7851
Standard Multiple Linear Regression	6.9634	3.5837
Lasso Regression	7.0708	3.5984
Ridge Regression	7.0667	3.5930
GAM Regression	6.6191	3.5502

**Note.** The MSE of predicted distributions represents the mean squared error between the model-predicted and observed distributions of log(claim amounts) for the test subgroup (*car age* = 6, *driver age* = 39).

**Fig 10:**
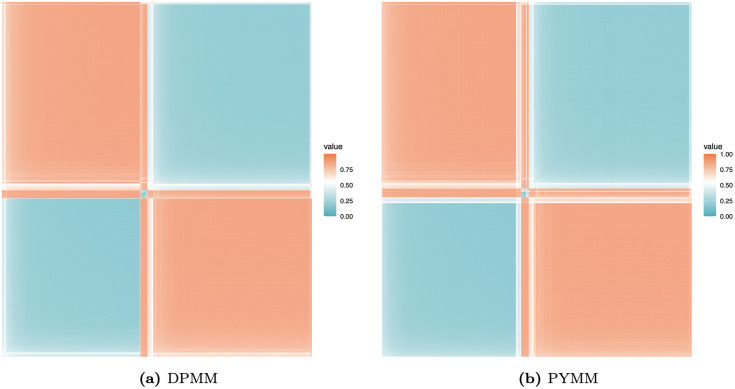
Heat maps of the dissimilarity matrices showing clustering structure for the Belgian motor claims severity dataset.

Fig 10 shows the posterior clustering heat maps for the Belgian motor claims severity data based on the dissimilarity matrix obtained under the two BNP models. The heat maps suggest approximately three clusters, indicating distinct latent severity groups in the Belgian portfolio. The presence of multiple severity clusters indicates that claim amounts are not well described by a single normal regression, highlighting the BNP models’ ability to adapt to distinct severity patterns in the data.

## 7 Discussion

For both claims frequency and severity, the proposed BNP regression models (DPMM and PYMM) show clear advantages over standard approaches. They yield posterior predictive distributions that closely match the empirical distributions of the holdout test subgroups, whereas the traditional parametric models (standard Poisson regression for frequency and multiple linear regression for log-severity) and the penalized or semi-parametric regression models (Lasso, Ridge, and GAM regressions) display noticeably poorer fit, based on the mean squared error (MSE) of the predicted distributions and visual analysis of the predictive distributions. In terms of predictive accuracy, the BNP models reduce the MSE by roughly one half for the French claims data, relative to the standard Poisson regression (frequency) and multiple linear regression (severity) baseline models, indicating substantial gains in predictive performance and a more realistic representation of the underlying claim behavior.

Assessment of predictive performance indicates that PYMM and DPMM have broadly similar accuracy. This similarity is likely attributable to the fact that the observed data in this application are not strongly heavy-tailed. The Pitman–Yor process prior is designed to accommodate heavier tails in the mixing distribution than the Dirichlet process, and we expect that the relative advantage of PYMM over DPMM would become more pronounced in applications where the underlying loss distribution exhibits a heavier tail than exponential, such as certain commercial liability or catastrophe-exposed portfolios.

Beyond predictive performance, the BNP regression models also provide outputs that can be interpreted and applied in actuarial practice. For example, in the French claims frequency data, the clustering analysis indicates that, once car age and driver age are included, the portfolio is fairly homogeneous: most policies have similar expected numbers of claims, and there is no strong evidence of distinct high- and low-frequency risk classes. This suggests that a single Poisson regression model may be adequate for describing the average number of claims with these covariates alone. Nevertheless, the BNP approach still improves on the standard Poisson regression model by flexibly capturing overdispersion and departures from the Poisson assumption in certain parts of the portfolio, rather than by uncovering clearly separated clusters. As a result, the BNP frequency models offer a richer description of the distribution of the number of claims and more accurate predictions for pricing and risk management.

For the French claims severity data, the clustering structure has particularly clear consequences for portfolio management. The heat maps and scatter plots indicate three latent severity clusters, corresponding to low-cost, medium-sized, and large claims, learned automatically by the BNP models. From an actuarial standpoint, the large-claims group is especially important, as it largely determines reinsurance needs and capital requirements, whereas the lower two groups are more closely linked to day-to-day pricing decisions. In practice, insurers could use the posterior cluster allocations to identify policies in the large-claims group and adjust underwriting rules, deductibles, or limits accordingly. Thus, the BNP regression results support both technical pricing (through improved predictive distributions) and more informed risk segmentation (through the induced clustering structure).

This work suggests several directions for future research. Methodologically, while we modeled frequency and severity separately, developing a joint BNP model that captures the dependence between claim numbers and claim amounts (e.g., via dependence structures such as copulas), and thus models aggregate claims directly, remains an important and challenging direction. It would also be of interest to construct hierarchical BNP models that allow for multi-level structure (e.g., policies nested within regions). From a computational perspective, exploring faster and scalable MCMC algorithms for these BNP regression models would be a valuable step toward real-time actuarial applications and could make BNP regression more accessible for routine pricing exercises on large portfolios. Finally, further applications to other lines of business and markets, along with systematic comparisons to alternative machine learning and Bayesian machine learning methods, would help clarify where BNP regression offers the greatest practical value for actuaries in pricing, risk management, and capital modeling.

## Supporting information

S1 Appendix Laplace approximation. (PDF)

S1 Table Summary of the main symbols used throughout the paper. (PDF)

## References

[1] Tse YK. Nonlife Actuarial Models: Theory, Methods and Evaluation. International Series on Actuarial Science. Cambridge University Press; 2009.

[2] Frees EW. Regression Modeling with Actuarial and Financial Applications. International Series on Actuarial Science. Cambridge University Press; 2009.

[3] Garrido J, Genest C, Schulz J. Generalized linear models for dependent frequency and severity of insurance claims. Insurance: Mathematics and Economics. 2016;70:205–15. doi: 10.1016/j.insmatheco.2016.06.006

[4] Fung TC, Tzougas G, Wüthrich MV. Mixture Composite Regression Models with Multi-type Feature Selection. North American Actuarial Journal. 2022;27(2):396–428. doi: 10.1080/10920277.2022.2099426

[5] Klein N, Denuit M, Lang S, Kneib T. Nonlife ratemaking and risk management with Bayesian generalized additive models for location, scale, and shape. Insurance: Mathematics and Economics. 2014;55:225–49. doi: 10.1016/j.insmatheco.2014.02.001

[6] Denuit M, Lang S. Non-life rate-making with Bayesian GAMs. Insurance: Mathematics and Economics. 2004;35(3):627–47. doi: 10.1016/j.insmatheco.2004.08.001

[7] Reck L, Schupp J, Reuß A. Identifying the determinants of lapse rates in life insurance: an automated Lasso approach. Eur Actuar J. 2022;13(2):541–69. doi: 10.1007/s13385-022-00325-1

[8] Laporta AG, Levantesi S, Petrella L. Neural networks for quantile claim amount estimation: a quantile regression approach. Ann actuar sci. 2023;18(1):30–50. doi: 10.1017/s1748499523000106

[9] Delong Ł, Lindholm M, Wüthrich MV. Gamma Mixture Density Networks and their application to modelling insurance claim amounts. Insurance: Mathematics and Economics. 2021;101:240–61. doi: 10.1016/j.insmatheco.2021.08.003

[10] Zhang Y, Ji L, Aivaliotis G, Taylor C. Bayesian CART models for insurance claims frequency. Insurance: Mathematics and Economics. 2024;114:108–31. doi: 10.1016/j.insmatheco.2023.11.005

[11] Su X, Bai M. Stochastic gradient boosting frequency-severity model of insurance claims. PLoS One. 2020;15(8):e0238000. doi: 10.1371/journal.pone.0238000 32866182 PMC7458339

[12] Clemente C, Guerreiro GR, Bravo JM. Modelling Motor Insurance Claim Frequency and Severity Using Gradient Boosting. Risks. 2023;11(9):163. doi: 10.3390/risks11090163

[13] Hartman B, Dahl D. Bayesian Nonparametric Regression for Diabetes Deaths. Actuarial Research Clearing House. 2010;20101.

[14] Hong L, Martin R. A Flexible Bayesian Nonparametric Model for Predicting Future Insurance Claims. North American Actuarial Journal. 2017;21(2):228–41. doi: 10.1080/10920277.2016.1247720

[15] Hong L, Martin R. Dirichlet process mixture models for insurance loss data. Scandinavian Actuarial Journal. 2017;2018(6):545–54. doi: 10.1080/03461238.2017.1402086

[16] Fellingham GW, Kottas A, Hartman BM. Bayesian nonparametric predictive modeling of group health claims. Insurance: Mathematics and Economics. 2015;60:1–10. doi: 10.1016/j.insmatheco.2014.10.011

[17] Richardson R, Hartman B. Bayesian nonparametric regression models for modeling and predicting healthcare claims. Insurance: Mathematics and Economics. 2018;83:1–8. doi: 10.1016/j.insmatheco.2018.06.002

[18] Huang Y, Meng S. A Bayesian nonparametric model and its application in insurance loss prediction. Insurance: Mathematics and Economics. 2020;93:84–94. doi: 10.1016/j.insmatheco.2020.04.010

[19] Ferguson TS. A Bayesian Analysis of Some Nonparametric Problems. Ann Statist. 1973;1(2). doi: 10.1214/aos/1176342360

[20] Sethuraman J. A constructive definition of Dirichlet priors. Statistica Sinica. 1994;:639–50.

[21] Neal RM. Markov Chain Sampling Methods for Dirichlet Process Mixture Models. Journal of Computational and Graphical Statistics. 2000;9(2):249–65. doi: 10.1080/10618600.2000.10474879

[22] Antoniak CE. Mixtures of Dirichlet Processes with Applications to Bayesian Nonparametric Problems. Ann Statist. 1974;2(6). doi: 10.1214/aos/1176342871

[23] Escobar MD, West M. Bayesian Density Estimation and Inference Using Mixtures. Journal of the American Statistical Association. 1995;90(430):577–88. doi: 10.1080/01621459.1995.10476550

[24] Pitman J, Yor M. The two-parameter Poisson-Dirichlet distribution derived from a stable subordinator. Ann Probab. 1997;25(2). doi: 10.1214/aop/1024404422

[25] Ishwaran H, James LF. Gibbs Sampling Methods for Stick-Breaking Priors. Journal of the American Statistical Association. 2001;96(453):161–73. doi: 10.1198/016214501750332758

[26] Teh YW. A hierarchical Bayesian language model based on Pitman–Yor processes. In: Proceedings of the 21st International Conference on Computational Linguistics and 44th Annual Meeting of the Association for Computational Linguistics. Sydney, Australia: Association for Computational Linguistics; 2006. p. 985-92. Available from: https://aclanthology.org/P06-1124/. doi:10.3115/1220175.1220299

[27] Fall MD, Barat É. Gibbs sampling methods for Pitman–Yor mixture models [Preprint]; 2014. Available from: https://hal.science/hal-00740770

[28] Lijoi A, Mena RH, Prunster I. Bayesian Nonparametric Estimation of the Probability of Discovering New Species. Biometrika. 2007;94(4):769–86. doi: 10.1093/biomet/asm061

[29] Roberts GO, Rosenthal JS. Examples of Adaptive MCMC. Journal of Computational and Graphical Statistics. 2009;18(2):349–67. doi: 10.1198/jcgs.2009.06134

[30] Hannah LA, Blei DM, Powell WB. Dirichlet Process Mixtures of Generalized Linear Models. Journal of Machine Learning Research. 2011;12(54):1923–53.

[31] Dutang C, Charpentier A. CASdatasets: Insurance datasets. 2025. doi: 10.57745/P0KHAG

[32] Information Systems and Wake Forest University. WFU High Performance Computing Facility. Wake Forest University; 2021. Available from: https://hpc.wfu.edu

[33] Plummer M, Best N, Cowles K, Vines K. CODA: Convergence Diagnosis and Output Analysis for MCMC. R News. 2006;6(1):7–11.

