# Supplementary material for: Modeling insurance claims using Bayesian nonparametric regression
Source: PLoS One. 2026 Apr 10;21(4):e0346734. doi: 10.1371/journal.pone.0346734 (PMC13068283; doi:10.1371/journal.pone.0346734)
Supplement: S1 Appendix — (PDF) [file pone.0346734.s001.pdf]

S1 Appendix. Laplace approximation.

Let $h(\boldsymbol{\beta}_{n+1})$ be a smooth function of the $(k+1)$-dimensional parameter $\boldsymbol{\beta}_{n+1}$, and consider integrals of the form

$$I \;=\; \int_{\mathbb{R}^{k+1}} \exp\{h(\boldsymbol{\beta}_{n+1})\}\, d\boldsymbol{\beta}_{n+1}.$$

In Section 3.2, such integrals arise when computing

$$\int f(y_{n+1} \mid \boldsymbol{\beta}_{n+1})\, g_0(\boldsymbol{\beta}_{n+1})\, d\boldsymbol{\beta}_{n+1} = \frac{(2\pi)^{\frac{-(k+1)}{2}}}{y_{n+1}!} \int \exp\{h(\boldsymbol{\beta}_{n+1})\}\, d\boldsymbol{\beta}_{n+1},$$

where $h(\boldsymbol{\beta}_{n+1})$ is defined as

$$h(\boldsymbol{\beta}_{n+1}) = -t_{n+1} e^{\boldsymbol{x}_{n+1}^T \boldsymbol{\beta}_{n+1}} + y_{n+1}(\log(t_{n+1}) + \boldsymbol{x}_{n+1}^T \boldsymbol{\beta}_{n+1}) - \frac{1}{2}\boldsymbol{\beta}_{n+1}^T \boldsymbol{\beta}_{n+1}.$$

Quadratic expansion

Let $\hat{\boldsymbol{\beta}}_{n+1}$ denote the maximizer of h, i.e.

$$\nabla h(\hat{\boldsymbol{\beta}}_{n+1}) = \mathbf{0}.$$

A second-order Taylor expansion of h around $\hat{\boldsymbol{\beta}}_{n+1}$ gives

$$h(\boldsymbol{\beta}_{n+1}) \approx h(\hat{\boldsymbol{\beta}}_{n+1}) - \frac{1}{2}(\boldsymbol{\beta}_{n+1} - \hat{\boldsymbol{\beta}}_{n+1})^T [\,-\nabla^2 h(\hat{\boldsymbol{\beta}}_{n+1})](\boldsymbol{\beta}_{n+1} - \hat{\boldsymbol{\beta}}_{n+1}),$$

where $\nabla^2 h$ is the Hessian matrix of second derivatives. Because $\hat{\boldsymbol{\beta}}_{n+1}$ is a maximizer, $-\nabla^2 h(\hat{\boldsymbol{\beta}}_{n+1})$ is positive definite.

Define

$$\hat{\Sigma} = [\,-\nabla^2 h(\hat{\boldsymbol{\beta}}_{n+1})]^{-1},$$

which serves as a local covariance matrix in the Laplace approximation.

Approximate evaluation of the integral

Using the quadratic expansion, the integrand becomes

$$\exp\{h(\boldsymbol{\beta}_{n+1})\} \approx \exp\{h(\hat{\boldsymbol{\beta}}_{n+1})\} \exp\left\{-\frac{1}{2}(\boldsymbol{\beta}_{n+1} - \hat{\boldsymbol{\beta}}_{n+1})^T \hat{\Sigma}^{-1}(\boldsymbol{\beta}_{n+1} - \hat{\boldsymbol{\beta}}_{n+1})\right\},$$

which is proportional to a multivariate normal density with mean $\hat{\boldsymbol{\beta}}_{n+1}$ and covariance $\hat{\Sigma}$. Therefore,

$$I \approx \exp\{h(\hat{\boldsymbol{\beta}}_{n+1})\} \int_{\mathbb{R}^{k+1}} \exp\left\{-\frac{1}{2}(\boldsymbol{\beta}_{n+1} - \hat{\boldsymbol{\beta}}_{n+1})^T \hat{\Sigma}^{-1}(\boldsymbol{\beta}_{n+1} - \hat{\boldsymbol{\beta}}_{n+1})\right\} d\boldsymbol{\beta}_{n+1}.$$

The remaining integral is the normalizing constant of a $(k+1)$-dimensional multivariate normal distribution:

$$\int_{\mathbb{R}^{k+1}} \exp\left\{-\frac{1}{2}(\boldsymbol{\beta} - \hat{\boldsymbol{\beta}})^T \hat{\Sigma}^{-1}(\boldsymbol{\beta} - \hat{\boldsymbol{\beta}})\right\} d\boldsymbol{\beta} = (2\pi)^{\frac{k+1}{2}} |\hat{\Sigma}|^{\frac{1}{2}}.$$

Thus, the Laplace approximation to I is

$$I \;\approx\; \exp\{h(\hat{\boldsymbol{\beta}}_{n+1})\}(2\pi)^{\frac{k+1}{2}} |\hat{\Sigma}|^{\frac{1}{2}}.$$

Application to the predictive distribution

In Section 3.2, this result yields the approximation

$$\begin{aligned}\int f(y_{n+1} \mid \boldsymbol{\beta}_{n+1})\, g_0(\boldsymbol{\beta}_{n+1})\, d\boldsymbol{\beta}_{n+1} &= \frac{(2\pi)^{\frac{-(k+1)}{2}}}{y_{n+1}!} \int \exp\left(h(\boldsymbol{\beta}_{n+1})\right) d\boldsymbol{\beta}_{n+1} \\ &\approx \frac{(2\pi)^{\frac{-(k+1)}{2}}}{y_{n+1}!} \exp\left(h(\hat{\boldsymbol{\beta}}_{n+1})\right)(2\pi)^{\frac{k+1}{2}} |\hat{\Sigma}|^{\frac{1}{2}} \\ &\approx \frac{1}{y_{n+1}!} \exp\left(h(\hat{\boldsymbol{\beta}}_{n+1})\right)|\hat{\Sigma}|^{\frac{1}{2}},\end{aligned}$$

which is substituted into the expression for the posterior predictive distribution of y_{n+1}.
