# Supplementary material for: Modeling insurance claims using Bayesian nonparametric regression
Source: PLoS One. 2026 Apr 10;21(4):e0346734. doi: 10.1371/journal.pone.0346734 (PMC13068283; doi:10.1371/journal.pone.0346734)
Supplement: S1 Table — (PDF) [file pone.0346734.s002.pdf]

S1 Table. Summary of the main symbols used throughout the paper.

Symbol	Description
$\mathrm{DP}(\alpha, G_0)$	Dirichlet process prior with precision α and base distribution G_0
$\mathrm{PY}(d, \alpha, G_0)$	Pitman–Yor process prior with discount d and strength α
G_0	Base probability distribution of the DP/PY prior
α	Precision (strength) parameter of the DP/PY prior
d	Discount parameter of the PY prior
G	Random probability distribution drawn from a DP or PY
K_n	Number of distinct components (clusters) among n observations
c_i	Cluster label associated with observation i
$\boldsymbol{\beta}_j^*, \boldsymbol{\theta}_j^*$	Unique parameter values for cluster j
$\boldsymbol{\phi}_c$	Distinct parameter associated with cluster c
n	Number of insurance policies (training observations)
k	Number of covariates (excluding the intercept)
i	Index for policies (observations)
$\boldsymbol{x}_i = (1, x_{i1}, \ldots, x_{ik})^T$	Covariate vector for policy i
y_i	Response for policy i (number of claims or log(claim amount))
z_i	Claim amount for policy i (severity model)
t_i	Exposure for policy i (frequency model)
$y_{n+1}, \boldsymbol{x}_{n+1}$	Future response and covariate vector
σ_i^2	Error variance for policy i (severity model)
$\boldsymbol{\beta}_i = (\beta_{i0}, \ldots, \beta_{ik})^T$	Regression coefficient vector for policy i
$\boldsymbol{\theta}_i = (\boldsymbol{\beta}_i, \sigma_i^2)$	Regression parameter vector for policy i (severity model)
$G_{0_{\boldsymbol{\beta}_i \mid \sigma_i^2}}$	Conditional base distribution for $\boldsymbol{\beta}_i \mid \sigma_i^2$ (severity model)
$G_{0_{\sigma_i^2}}$	Base distribution for σ_i^2 (severity model)
n_0	Scale parameter in the covariance of $G_{0_{\boldsymbol{\beta}_i \mid \sigma_i^2}}$
a, b	Shape and scale hyperparameters of Inverse-Gamma(a, b)
M	Number of MCMC posterior samples
$K_n^{(m)}$	Number of clusters at MCMC iteration m
$\boldsymbol{\beta}_i^{(m)}, \boldsymbol{\theta}_i^{(m)}$	Posterior samples at iteration m
$f(\cdot)$	Likelihood or predictive probability density/mass function
$g_0(\cdot)$	Density of the base distribution G_0

Note. Bold symbols (e.g., $\boldsymbol{x}_i$, $\boldsymbol{\beta}_i$) denote vectors; superscripts (m) index MCMC iterations.
